# Cholecystoduodenal Fistula: A Case Series of an Unusual Complication of Gallstone Diseases

**DOI:** 10.7759/cureus.31651

**Published:** 2022-11-18

**Authors:** P. Senthil Kumar, Sakthivel Harikrishnan

**Affiliations:** 1 Surgical Gastroenterology, Government Villupuram Medical College, Villupuram, IND; 2 Surgical Gastroenterology and Liver Transplant, Government Stanley Medical College, Chennai, IND

**Keywords:** enterolithotomy, internal biliary fistula, gallstones complication, gallstone diseases, cholecystoenteric fistula, cholecystoduodenal fistula

## Abstract

The usual complications of gallstone diseases are acute cholecystitis, choledocholithiasis, cholangitis, and acute pancreatitis. Most of the patients who present with these complications have a prior history suggestive of gallstone diseases. Cholecystoenteric fistula is a very uncommon complication of gallstone disease, and many patients do not have a previous history suggestive of biliary pathology. Gallstone ileus is a mechanical cause of intestinal obstruction due to the passage of a large stone through the cholecystoenteric fistula. These patients present with vague clinical signs and symptoms and non-specific laboratory abnormalities; hence, a high index of suspicion is needed for early diagnosis and management of the same. Once diagnosed, controversies exist in their appropriate surgical management. We present a series of four cases of cholecystoduodenal fistula, two patients presenting with gallstone ileus, and two patients presenting with cholangitis and their successful surgical management.

## Introduction

The biliary fistula can be external or internal. External biliary fistulas are often seen due to surgical or percutaneous biliary interventions. Internal biliary fistula is an abnormal communication between the biliary system and the internal organs and is classified as biliobiliary, bilioenteric, and bronchobiliary. Chronic cholecystitis is the most common cause of internal biliary fistula [1]. The other uncommon causes of internal biliary fistulas are peptic ulcer disease and malignancies of the stomach, pancreas, colon, and duodenum.

The anatomical types of internal biliary fistulas are cholecystoduodenal, cholecystoenteric, cholecystogastric, cholecystocholedochal, and cholecystoduodenocolic. It represents 54-76% of all types of internal fistulas [2]. Gallstone ileus is an uncommon cause of mechanical small bowel obstruction in which a large stone from the gall bladder (GB) passes through the fistula into the small intestine and gets impacted, most commonly in the terminal ileum to cause small bowel obstruction. Patients with an internal biliary fistula mostly present with non-specific signs and symptoms and a high index of suspicion is needed to diagnose this preoperatively. Due to the sophisticated modern imaging techniques, more cases of internal biliary fistulas are being diagnosed, and managing the same becomes challenging. It is an uncommon complication of calculous biliary disease with literature evidence of only case reports and case series. Here, we report a series of four cases of cholecystoduodenal fistula presenting as gallstone ileus in two patients and as cholangitis in the other two patients and the surgical technique used in the management of these patients.

## Case presentation

All patients underwent routine preoperative workups, which included a complete blood count, liver and renal function tests, and imaging. The imaging studies included an ultrasound abdomen and a contrast-enhanced CT abdomen. The preoperative characteristics of the four patients with cholecystoduodenal fistula are depicted in Table 1.

**Table 1:** Preoperative characteristics of the patients with cholecystoduodenal fistula. DM: diabetes mellitus, HTN: hypertension, COPD: chronic obstructive pulmonary disease, SLE: systemic lupus erythematosus, SGOT: serum glutamic-oxaloacetic transaminase, SGPT: serum glutamic-pyruvic transaminase.

Characters	Patient 1	Patient 2	Patient 3	Patient 4
Age and sex	75/Male	58/Female	43/Male	45/Female
Clinical symptoms	Abdominal distension	Colicky pain abdomen	Pain abdomen	Pain upper abdomen
	Obstipation	Vomiting	Fever with chills	Vomiting
	Fever	Obstipation	Fatigue	Fever
Clinical signs	Massive abdominal distension	Abdominal distension	Right hypochondrial tenderness	Right hypochondrial tenderness
Comorbidities	COPD/DM/HTN	DM/HTN	Nil	SLE on steroids
WBC count	15400	14000	17000	16200
Liver function tests				
Total bilirubin (mg/dl)	1.2	1	3	0.8
Direct bilirubin (mg/dl)	0.4	0.6	2.4	0.4
SGOT (IU/L)	16	20	20	25
SGPT (IU/L)	25	36	35	15
Alkaline phosphatase (IU/L)	110	100	200	109

The mean age of the patients with cholecystoduodenal fistula was 55.25 years (range 43-75 years). Two patients presented with features of intestinal obstruction, and two patients presented with features of cholangitis. Two patients who presented with intestinal obstruction had diabetes and hypertension as comorbid conditions. One of the patients who presented with cholangitis was a known case of SLE on steroids. All four patients had leucocytosis. Only one patient with cholangitis had altered liver function tests with slightly raised bilirubin and alkaline phosphatase levels. The imaging characteristics of all four patients are depicted in Table 2.

**Table 2:** Imaging characteristics of the patients with cholecystoduodenal fistula. CECT: contrast-enhanced computed tomography, MRCP: magnetic resonance cholangiopancreatography, USG: ultrasonography.

Imaging	Patient 1	Patient 2	Patient 3	Patient 4
X-ray abdomen	Dilated small bowel loops. More than five air-fluid levels	Dilated small bowel loops. Multifaceted stones in the terminal ileum	Not taken	Not taken
USG abdomen	Edematous and dilated small bowel loops with sluggish peristalsis. Contracted gall bladder	Distended bowel loops	Air in the gall bladder lumen. Gall bladder calculi pneumobilia	Gall bladder contracted with luminal air. Pneumobilia portal lymph nodes. Common bile duct 5 mm
CECT abdomen	Pneumobilia, cholecystoduodenal fistula, dilated small bowel loops, intraluminal abnormal cholecystoduodenal, shadow in the terminal ileum fistula suggestive of gallstone ileus.	Gall bladder calculus, common bile duct dilated, and pneumobilia. Stenosed cystic duct: two large faceted stones in the terminal ileum producing intestinal obstruction. Juxta ampullary diverticulum present.	Air in the fundus of gallbladder pneumobilia	Air in the lumen of gallbladder pneumobilia
MRCP	Not available	Not available	Gallbladder calculi, cholecystoduodenal fistula, common bile duct: normal	Not available

**Figure 1:**
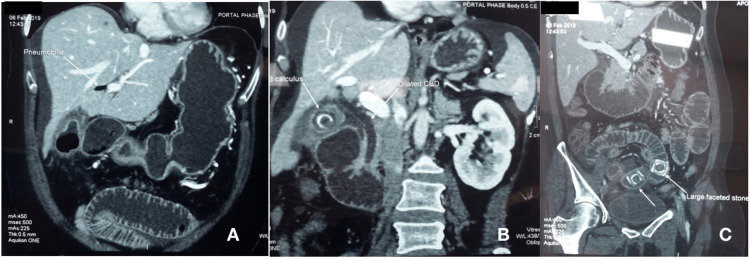
Radiological findings of patient no. 2. (A) White arrow shows the pneumobilia, (B) white arrow shows the contracted gall bladder with gallstone, and (C) shows dilated small bowel loops with large faceted stones in the terminal ileum.

The two patients with intestinal obstruction had dilated, edematous small bowel loops and the gall bladder could not be imaged as it was obscured by the bowel gas. A contrast-enhanced CT scan was done in all four patients, which showed gall bladder luminal air and pneumobilia characteristic of a cholecystoenteric fistula. In the two patients who presented with intestinal obstruction, a CT scan showed intraluminal stones in the terminal ileum with dilated proximal small bowel loops, pneumobilia, and atrophic gall bladder, suggestive of Rigler's triad of gallstone ileus (Figure 1). One of these patients showed serial migration of the stone in the CT scan. Gallbladder stones, the air in the lumen of the gall bladder, and pneumobilia were the cardinal findings in the patients who presented with cholangitis. There was no significant intra- or extrahepatic biliary radical dilatation in any patients. Only one patient underwent MRCP, which delineated the cholecystoduodenal fistula.

All four patients were resuscitated initially with fluids and intravenous antibiotics and then surgically managed. The intraoperative findings and the surgical characteristics are depicted in Table 3.

**Table 3:** Intraoperative findings of the patients with cholecystoduodenal fistula. CBD: common bile duct, GB: gall bladder, IC: ileocecal.

S. no	Duration of hospital stay (days)	Age/sex	Diagnosis	Operative findings	Surgery	Duration (mins)	Blood loss (ml)
Patient 1	12	75/M	Gallstone ileus	3.5 cm of obstructing calculus in the terminal ileum, about 15 cm proximal to the ileocecal junction.	Enterolithotomy	60	40
Patient 2	10	58/F	Gallstone ileus	Small bowel obstruction due to two faceted stones impacted in the ileum 50 cm proximal to the IC junction. The proximal ileum and jejunum dilated. GB calculus with chronic cholecystitis with cholecystoduodenal fistula.	Enterolithotomy and cholecystectomy. Division of the cholecystoduodenal fistula. Closure of the duodenum in two layers.	120	60
Patient 3	7	63/M	Mild cholangitis secondary to cholecystoduodenal fistula	Dense sub-hepatic adhesions. Contracted GB fistula is present between the fundus of the GB and the duodenum.	Open cholecystectomy. Resection of the fistulous tract. Primary closure of the duodenum in two layers.	90	150
Patient 4	6	45/F	Cholecystoduodenal fistula	Contracted GB. Omentum and duodenum adherent to the GB cholecystoduodenal fistula. CBD normal.	Lap converted to open cholecystectomy. Closure of the duodenal rent with an omental patch.	100	90

**Figure 2:**
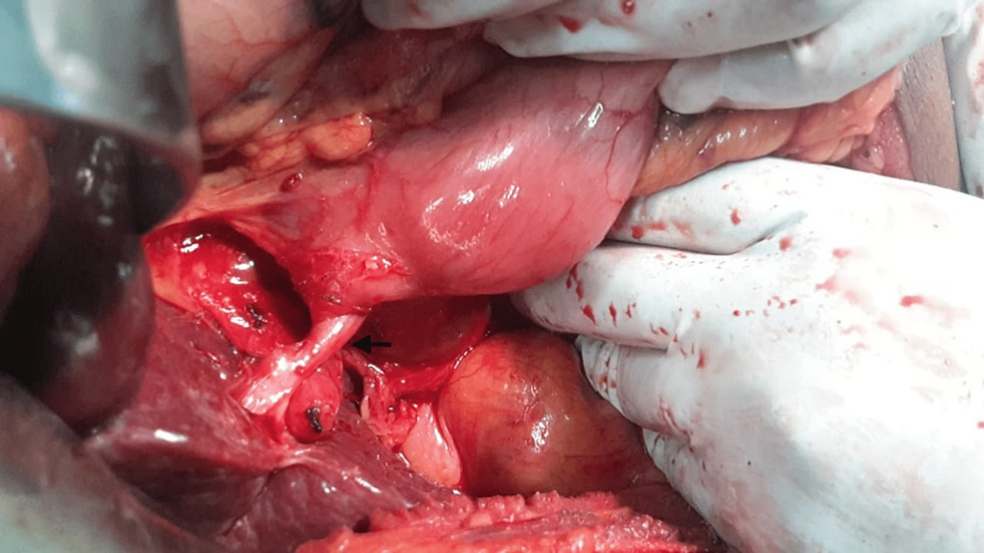
Intraoperative picture of patient no. 3 showing contracted gall bladder with cholecystoduodenal fistula (black arrow).

Of the two patients who presented with gallstone ileus, one underwent a definitive procedure that included enterolithotomy, cholecystectomy, and closure of the cholecystoduodenal fistula (Figure 2). In view of the patient's age and comorbid conditions, the other patient underwent enterolithotomy alone to relieve the bowel obstruction. The two patients who presented with cholangitis underwent cholecystectomy, excision of the fistulous tract, and repair of the duodenal wall. All the patients underwent an open surgical procedure. The blood loss was minimal, and the patients were discharged within two weeks. There was no mortality in our series. One of the patients who was operated on for gallstone ileus developed wound dehiscence, which was managed conservatively with daily dressings. At one-year follow-up, all patients remained asymptomatic, including those who underwent only enterolithotomy for gallstone ileus.

## Discussion

Spontaneous internal biliary fistula is usually caused by chronic diseases of the biliary tract, either cholelithiasis or choledocholithiasis. It is most often seen in patients of advancing age with a female preponderance, and a history of gallstone disease is not always present in all patients [3]. In the presence of concomitant biliary tract obstruction, gallstone erosion to nearby viscera occurs in a chronically inflamed gall bladder. The most common types of spontaneous internal biliary fistulas are cholecystoduodenal (70%), followed by cholecystocolonic (14%) and cholecystogastric (6%) [4]. All patients reported in our series had cholecystoduodenal fistula.

Various imaging modalities are helpful in identifying the cholecystoenteric fistula and gallstone ileus. The plain abdominal radiography clearly shows the Rigler's triad (pneumobilia, ectopic radiopaque gallstones, and dilatation of bowel loops) in 17-87% of patients. Ultrasound of the abdomen can highlight pneumobilia, residual gallstones, and ectopic gallstones. The CT abdomen has 93% sensitivity and 100% specificity for diagnosing gallstone ileus. Apart from diagnosing pneumobilia, a CT scan can delineate the site of intestinal obstruction, the size of the stone, and the location of the fistula. MRCP can identify pneumobilia but has a decreased sensitivity in identifying gallstone ileus [5]. In some patients, the fistula can be identified only by invasive techniques like endoscopic retrograde cholangiopancreatography (ERCP), in which case therapeutic sphincterotomy can be done, which might heal the fistula [6].

The fistula formation is a "fortunate accident" to relieve the obstruction. Once the fistulous tract is established and if a sufficiently large-sized stone passes through the fistula, the patients can present with intestinal obstruction in the form of gallstone ileus, whereas other patients can present with cholangitis or non-specific symptoms in the background of calculous biliary disease [7]. Thus, patients with spontaneous internal biliary fistulas can present as obstructive or non-obstructive types.

The obstructive type of spontaneous internal biliary fistula, also known as gallstone ileus, accounts for less than 2% of the causes of small bowel obstruction but occurs increasingly in the elderly population. The stone that passes through the fistula most commonly passes distally. If the stone is about 2.5 cm in size, it is sufficiently large to get impacted in the ileum and, less commonly, in the jejunum and the sigmoid colon. Very rarely, the stone may pass proximally to cause duodenal obstruction [8]. As the stone migrates, it may cause intermittent obstruction described as migratory or tumbling obstruction. All the patients in our series had the stone impacted in the terminal ileum. Patients with the obstructive type are managed with a laparotomy, and the stone is usually milked to the healthy segment of the bowel to be removed by an enterolithotomy. A thorough bowel examination is to be done to search for any missed stones that might cause recurrent obstruction. The definitive management, which includes cholecystectomy and excision of the fistulous tract, can be done based on the patient's condition [4,9].

Enterolithotomy alone and enterolithotomy plus cholecystectomy with excision of the fistulous tract are the two surgical strategies employed to manage a patient with gallstone ileus. There is no high-level evidence regarding which strategy is the best. Tan et al. reported 19 cases of gallstone ileus (12-enterolithotomy alone, 7-enterolithotomy plus cholecystectomy, and fistula closure). They concluded that enterolithotomy is safe in both low- and high-risk patients requiring a shorter operative time, less demanding technically, and no fistula-related complications in the future. He emphasized that relieving the obstruction is the mainstay of treatment in patients with gallstone ileus [10]. Doko et al. retrospectively analyzed 30 patients with gallstone ileus. They concluded that the complication occurrence is significantly higher with definitive fistula repair, and enterolithotomy should be the surgery of choice in gallstone ileus. The definitive repair should be exclusively reserved for highly selected patients with absolute indications like gangrene of the gall bladder wall, acute cholecystitis, and residual stones [11]. In our series, we did enterolithotomy alone for the 75-year-old male patient because of his multiple comorbidities. The other patient with gallstone ileus underwent definitive fistula management with no significant postoperative morbidity or mortality.

In the non-obstructive type, the patient usually presents with non-specific signs and symptoms and is identified intraoperatively during exploration for calculous biliary disease. Some patients, especially those with colonic fistula, can present with ascending cholangitis [12]. Those patients with cholangitis need fistula repair to avoid recurrent ascending cholangitis [13]. Two of the patients in our series presented with cholangitis features and managed with open cholecystectomy and definitive fistula repair.

Patients with cholecystoenteric fistula can be safely managed with laparoscopic procedures and don't necessitate conversion to open cholecystectomy in the present era. In high-volume and well-equipped centers, the cholecystoenteric fistula can be safely managed with the laparoscopic technique. Chowbey et al. managed 59/63 patients with cholecystoenteric fistulas laparoscopically. They prefer using an endostapler to transect the fistula rather than intracorporeal sutures of the duodenal defect to prevent contamination [14]. Due to multiple comorbidities and severe sub-hepatic adhesions, we decided to perform open cholecystectomy in all our patients.

## Conclusions

Cholecystoenteric fistula is a rare complication of symptomatic gallstone disease. However, there is no large case series available in the literature to validate the safe surgical approach in patients with gallstone ileus. Most of the reports advocate enterolithotomy alone in the obstructive type. The cholecystectomy and fistula repair can be done only if absolutely indicated. Cholecystectomy and fistula repair should be the preferred procedure in patients with septic complications of the non-obstructive type.
